# Multiparametric MRI quantitative metrics for grading and staging graves’ ophthalmopathy

**DOI:** 10.1186/s12880-026-02398-w

**Published:** 2026-05-09

**Authors:** Shaopeng Wu, Libin Yang, Yong Cai, Jixing Liang, Junping Wen, Honglei Huang, Shun Yu

**Affiliations:** 1 Department of Medical Imaging, Nanping First Hospital Affiliated to Fujian Medical University, Nanping, Fujian 353000 China; 2 Department of Radiology, Shengli Clinical Medical College of Fujian Medical University, Fujian Provincial Hospital, Fuzhou University Affiliated Provincial Hospital, 134 East Street, Fuzhou, Fujian 350001 China; 3 Department of Endocrinology, Shengli Clinical Medical College of Fujian Medical University, Fujian Provincial Hospital, Fuzhou University Affiliated Provincial Hospital, Fuzhou, Fujian 350001 China; 4 Fujian Provincial Key Laboratory of Medical Big Data Engineering, Fuzhou, China

**Keywords:** Graves' ophthalmopathy, Activity staging, Severity grading, Multiparametric magnetic resonance imaging, Joint model

## Abstract

**Objective:**

To explore the application of multiparametric magnetic resonance imaging (MRI) combined with clinical parameters in the assessment of Graves’ ophthalmopathy (GO).

**Methods:**

This retrospective study included 71 patients with GO (142 affected eyes) categorized into inactive (75) vs. active (67) and mild (70) vs. moderate-severe (72) subgroups. Clinical parameters were collected, and extraocular muscle (EOM) parameters [signal intensity ratio (SIR), T1 relaxation times (T1RT), T2 relaxation times (T2RT), and fat fraction (FF)] were measured (max/mean values). Predicted probabilities were derived from the generalized linear mixed model. Multivariate Logistic regression (based on the Akaike Information Criterion) was used to develop combined diagnostic models, and the marginal area under the receiver operating characteristic (ROC) curve (AUC) was employed to assess their efficacy.

**Results:**

Compared with the inactive and mild groups, the active and moderate-severe GO eyes showed higher mean EOM-SIR, mean EOM-T1RT, and mean/max EOM-T2RT, as well as lower mean/max EOM-FF and female proportion (all *P* < 0.05). The active group had a shorter disease duration, whereas the moderate-severe group had higher thyrotropin receptor antibody (TRAb) levels (*P* < 0.05). For differentiating GO activity and severity, the combined staging Model 3 (EOM-FFmax + EOM-T1RTmean + EOM-T2RTmean + sex + thyroid functional status) and grading Model 6 (EOM-FFmax + EOM-T1RTmean + EOM-T2RTmean + sex + TRAb + thyroid functional status) yielded the best diagnostic efficacy, with AUCs of 0.912 and 0.947, respectively.

**Conclusion:**

The combined model of quantitative multiparametric MRI and clinical parameters enables a more accurate assessment of GO activity and severity.

**Supplementary Information:**

The online version contains supplementary material available at 10.1186/s12880-026-02398-w.

## Introduction

Graves’ orbitopathy (GO) is a complex autoimmune disease, with smoking, thyroid dysfunction, and hypercholesterolemia as its major risk factors [1, 2]. Severe cases may lead to blindness [1, 2].For patients with active, moderate-severe GO, intravenous glucocorticoid pulse therapy is the first-line treatment, whereas inactive patients usually undergo surgical intervention [3]. Accurate grading and staging are vital for determining GO treatment strategies. The clinical activity score (CAS) stages GO activity, and severity is graded according to the 2021 European Group on GO (EUGOGO) criteria; however, both approaches rely on subjective assessments [3, 4].

In recent years, imaging technologies have become crucial in the diagnosis and treatment of GO. Orbital ultrasonography reveals eyeball protrusion and morphological changes in the extraocular muscles (EOMs); however, blurring of the rims of EOMs can cause measurement inaccuracies [5, 6]. Computed tomography (CT) shows orbital bony structures, EOM changes, eyeball protrusion, and intraorbital fat content; however, its ionizing radiation limits its long-term use [7]. Magnetic resonance imaging (MRI) stands out for the evaluation of EOM lesions. Its high resolution enables the clear visualization of subtle soft tissue changes behind the eye. Metrics such as the signal intensity ratio (SIR) and T2 relaxation time (T2RT) reflect EOM inflammation and edema [8–11]; T1 relaxation time (T1RT) is useful for assessing fibrosis and fatty infiltration [12, 13], and fat fraction (FF) quantifies fat content in EOMs [8, 10]. Despite the application of advanced MRI techniques, integrating quantitative multiparametric MRI metrics with clinical parameters for a comprehensive GO assessment requires further study.

This study aimed to explore the application of multiparametric MRI combined with clinical parameters to assess GO activity and severity.

## Materials and methods

### Study objectives

**Fig. 1:**
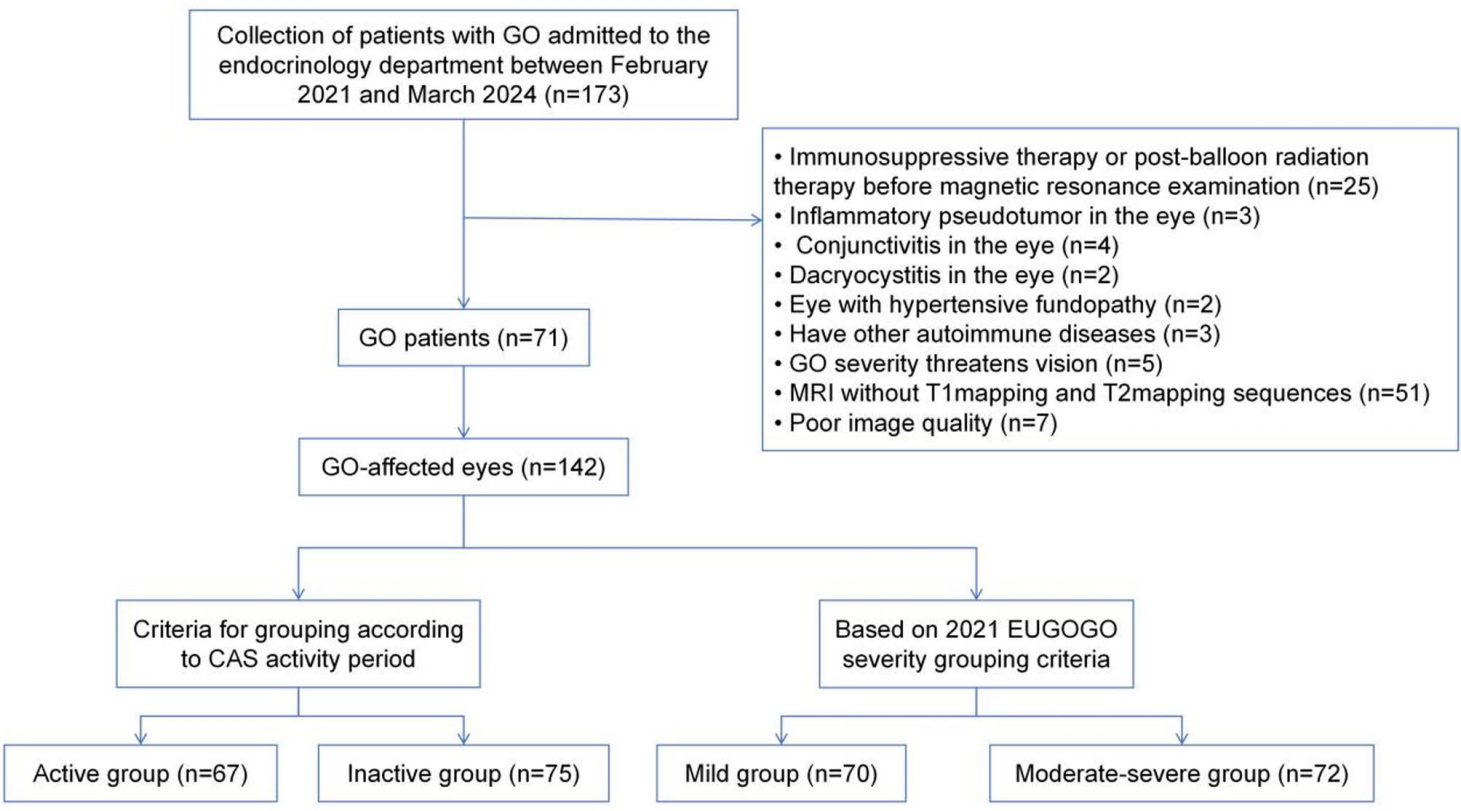
Flowchart of the study participant inclusion process. GO: Graves’ orbitopathy. CAS: Clinical Activity Score. EUGOGO: European Group on Graves’ Orbitopathy

This study enrolled 173 patients diagnosed with GO between February 2021 and March 2024 at the Endocrinology Department of Fujian Provincial Hospital. According to the inclusion and exclusion criteria, 71 GO patients were finally included, with each eye treated as a separate observation unit, totaling 142 affected eyes (Fig. 1). Inclusion criteria: Patients meeting the internationally recognized Bartal diagnostic criteria [14]. Exclusion criteria: (1) Prior immunosuppressive therapy or retrobulbar radiation therapy before MRI; (2) Other ocular diseases (e.g., inflammatory pseudotumor, orbital tumor); (3) Infectious eye conditions (e.g., conjunctivitis, dacryocystitis); (4) Hypertensive fundus lesions; (5) Coexisting autoimmune diseases; (6) Sight-threatening GO; (7) Absence of T1/T2 mapping sequences on MRI; (8) Poor-quality MRI images with artifacts.

### Clinical information and subgroups

Data on clinical parameters (sex, age, disease duration, smoking history, and thyroid functional status) were collected from all participants. Thyroid functional status parameters included free triiodothyronine (FT3), free thyroxine (FT4), thyroid-stimulating hormone (TSH), and thyrotropin receptor antibodies (TRAb). The Clinical Activity Score (CAS) comprised seven items [4]: (1) spontaneous retrobulbar pain, (2) pain with gaze or eye movement, (3) eyelid redness, (4) conjunctival redness, (5) eyelid edema, (6) bulbar conjunctival edema, and (7) lacrimal frenulum swelling. One clinical manifestation was recorded as one point, and the total score was 7. GO-affected eyes with a CAS ≥ 3 were classified as the active group, whereas those with a CAS < 3 were categorized as the inactive group. The GO severity was stratified into mild and moderate-severe groups according to the 2021 EUGOGO severity grading scale [3].

### MRI examinations

All patients underwent 3.0 T MRI scanning (MAGNETOM Prisma, Siemens Healthcare, Erlangen, Germany). The patient was placed in the supine position, with the head fixed in a 20-channel head coil. Patients were instructed to keep their eyes closed and gaze directed forward to restrict eye movement. The scanning field covered the superior and inferior orbital rims. Scanning sequences included coronal Dixon T2WI, T1 mapping, T2 mapping, as well as axial T1WI and Dixon T2WI. Detailed scanning parameters are listed in Table S1.

### Methods for MRI data measurement and processing

Coronal Dixon T2WI, T1 mapping, and T2 mapping raw data were processed through syngo.via a post-processing workstation, and pseudo-color maps of T1 mapping and T2 mapping were generated. The FF pseudo-color map was reconstructed based on the fat and water phase images of Dixon T2WI using the following Eqs. [15, 16]:

$$\eqalign{{\rm{FF}} & = {\rm{EOM}}\,{\rm{fat}}\,{\rm{signal}}\,{\rm{intensity}} \cr& /({\rm{EOM}}\,{\rm{fat}}\,{\rm{signal}}\,{\rm{intensity}} \cr& + {\rm{EOM}}\,{\rm{water}}\,{\rm{signal}}\,{\rm{intensity}}) \cr} $$

1. Measurements of EOM-SIR: Coronal Dixon T2WI water-phase images on the largest cross-sectional level of the EOMs (the 3rd or 4th slice posterior to the globe) were selected [10, 17]. Measurements were independently performed by two researchers with 3 and 5 years of imaging experience, respectively. Regions of interest (ROIs) were manually drawn along the outer margins of the four EOMs (superior rectus, inferior rectus, medial rectus, and lateral rectus). Signal intensities of the ipsilateral cerebral white matter were measured, and the SIR and extraocular muscle area (EMA) for each EOM were obtained (Fig. S1-A).
2. Measurement of EOM-FF, EOM-T1RT, EOM-T2RT: Coronal FF pseudo-color maps, T1 mapping pseudo-color maps, and T2 mapping pseudo-color maps were selected at the same level as the EOM-SIR measurements. Using the same measurement method as for EOM-SIR, the FF, T1RT, T2RT values, and EMAs were obtained for each of the four EOMs (Fig. S1-B-D).

EOM-SIRmax, EOM-FFmax, EOM-T1RTmax, and EOM-T2RTmax are the maximum values of the four corresponding EOMs. EOM-SIRmean, EOM-FFmean, EOM-T1RTmean, and EOM-T2RTmean were calculated as follows [15, 16]:

$$\eqalign{{\rm{EOM}}& \left({{\rm{SIR}},\,{\rm{FF}},\,{\rm{T}}1{\rm{RT}}\,{\rm{and}}\,{\rm{T}}2{\rm{RT}}} \right)\,{\rm{mean}} \cr& = \,({\rm{medial}}\,{\rm{rectus}}\,\left({{\rm{SIR}},\,{\rm{FF}},\,{\rm{T}}1{\rm{RT}}\,{\rm{and}}\,{\rm{T}}2{\rm{RT}}} \right) \cr& \times \,{\rm{medial}}\,{\rm{rectus}}\,{\rm{area}} + \,{\rm{lateral}}\,{\rm{rectus}} \cr& \left({{\rm{SIR}},\,{\rm{FF}},\,{\rm{T}}1{\rm{RT}}\,{\rm{and}}\,{\rm{T}}2{\rm{RT}}} \right)\, \times \,{\rm{lateral}}\,{\rm{rectus}}\,{\rm{area}} \cr& + {\rm{}}\,{\rm{superior}}\,{\rm{rectus}}\,\left({{\rm{SIR}},\,{\rm{FF}},\,{\rm{T}}1{\rm{RT}}\,{\rm{and}}\,{\rm{T}}2{\rm{RT}}} \right) \cr& \times \,{\rm{superior}}\,{\rm{rectus}}\,{\rm{area}} \cr& + \,{\rm{inferior}}\,{\rm{rectus}}\,\left({{\rm{SIR}},\,{\rm{FF}},\,{\rm{T}}1{\rm{RT}}\,{\rm{and}}\,{\rm{T}}2{\rm{RT}}} \right) \cr& \times \,{\rm{inferior}}\,{\rm{rectus}}\,{\rm{area}})/({\rm{medial}}\,{\rm{rectus}}\,{\rm{area}} \cr& + \,{\rm{lateral}}\,{\rm{rectus}}\,{\rm{area}} + \,{\rm{superior}}\,{\rm{rectus}}\,{\rm{area}} \cr& + \,{\rm{inferior}}\,{\rm{rectus}}\,{\rm{area}}) \cr} $$

### Statistical analysis

Statistical analyses were conducted using R 4.3 and Origin 2021. Continuous variables were tested for normality using the Shapiro-Wilk test and presented as mean ± SD for normally distributed data or median (Q1, Q3; interquartile range) for non-normal distributions. Intraclass and interclass correlation coefficients (ICC) were used to evaluate measurement agreement for repeated assessments by the same physician and between two physicians, respectively. Since each patient contributed two eyes, and the two eyes are correlated within individuals, the generalized linear mixed model was used for eye-level comparisons to account for within-patient correlation. Categorical variables were reported as n (%) and compared using the χ² test. Predicted probabilities were obtained from the generalized linear mixed model by integrating out the random effects, yielding population-averaged estimates. Variables with *P* < 0.1 in univariate analysis and those considered important based on clinical expertise were included in the multivariate Logistic regression, and independent predictors were selected via the Akaike Information Criterion (AIC) criterion to develop models for GO activity staging and severity grading. Diagnostic performance was assessed using the marginal area under the receiver operating characteristic curve (marginal AUC), along with the optimal cutoff value, sensitivity, and specificity derived from the predicted probabilities. For pairwise comparisons of AUCs, $\:\mathrm{D}\mathrm{e}\mathrm{L}\mathrm{o}\mathrm{n}\mathrm{g}{\prime\:}\mathrm{s}$ test was employed. *P* < 0.05 was considered statistically significant.

## Results

### Demographic and clinical characteristics

This study included 71 patients with GO. The participants’ age range was 21–75 years. There were 27 (38%) male patients and 44 (62%) female patients. Among them, 12 (17%) were smokers. In terms of thyroid function, 32 (45%) patients had normal thyroid function, 8 (11%) had subclinical hyperthyroidism, 20 (28%) had hyperthyroidism, and 11 (15%) had hypothyroidism. The median disease duration was 4 (2, 9) months. The median FT3 level was 5.10 (4.40, 7.01) pg/mL, FT4 was 18.00 (15.00, 23.00) ng/dL, TSH was 0.67 (0.01, 2.34) µIU/mL, and TRAb was 6.00 (3.00, 18.00) IU/L (Table S2).

### Results of intra- and inter-group correlation coefficient analysis

The intra-group and inter-group ICC of EOM-SIRmean, EOM-SIRmax, EOM-T1RTmean, EOM-T1RTmax, EOM-T2RTmean, and EOM-T2RTmax demonstrated good consistency. The ICC values ranged from 0.735 to 0.962 for intra-group ICC values and 0.751 to 0.951 for inter-group ICC values, respectively (Tables S3 and S4).

### Comparison of clinical and MRI parameters between active and inactive groups

Compared with the inactive group, the active group showed higher values for EOM-SIRmean, EOM-SIRmax, EOM-T1RTmean, EOM-T2RTmean, and EOM-T2RTmax, while presenting lower values for EOM-FFmean, EOM-FFmax, and female proportion (all *P* < 0.05). In addition, the active group had a shorter disease duration (*P* < 0.05). No significant differences between the groups were observed in smoking history, thyroid functional status, FT3, FT4, TSH, TRAb, and EOM-T1RTmax (all *P* > 0.05) (Table 1, Fig. S2).

**Table 1:** Comparison of clinical and MRI parameters between the active and inactive GO groups

Parameter	Inactive group (*n* = 75)	Active group (*n* = 67)	*P*
Sex (n, %)			< 0.001
Male	14 (19%)	40 (60%)	
Female	61 (81%)	27 (40%)	
Smoking (n, %)			0.061
+	8 (11%)	16 (24%)	
−	67 (89%)	51 (76%)	
Thyroid functional status (n, %)			0.058
Normal	35 (47%)	29 (43%)	
Subclinical hyperthyroidism	13 (17%)	3 (4.5%)	
Hyperthyroidism	17 (23%)	23 (34%)	
Hypothyroidism	10 (13%)	12 (18%)	
Disease duration (months)	6 (3, 12)	4 (2, 7)	0.042
FT3 (pg/mL)	4.90 (4.36, 7.63)	5.24 (4.440, 6.77)	0.787
FT4 (ng/dL)	17.44 (15.17, 24.03)	17.61 (15.170, 23.16)	0.747
TSH (µIU/mL)	0.80 (0.01, 1.98)	0.53 (0.01, 2.85)	0.646
TRAb (IU/L)	4.97 (2.98, 12.30)	7.07 (2.93, 21.32)	0.075
EOM-FFmean (%)	7.00 (5.00, 9.60)	4.90 (2.10, 6.70)	0.005
EOM-FFmax (%)	10.00 (8.00, 16.00)	7.00 (4.00, 11.00)	0.005
EOM-SIRmean	0.92 (0.80, 1.07)	1.21 (1.04, 1.34)	< 0.001
EOM-SIRmax	1.08 (0.95, 1.28)	1.42 (1.28, 1.58)	< 0.001
EOM-T1RTmean (ms)	1242.00 (1070.00, 1480.00)	1375.00 (1268.00, 1551.00)	< 0.001
EOM-T1RTmax (ms)	1646.00 (1484.00, 1758.00)	1723.00 (1480.00, 1850.00)	0.213
EOM-T2RTmean (ms)	87.00 (81.00, 97.00)	109.00 (96.00, 123.00)	< 0.001
EOM-T2RTmax (ms)	96.00 (89.00, 106.00)	125.00 (108.00, 148.00)	< 0.001

Note: P values are calculated from generalized mixed linear models. FT3: free triiodothyronine. FT4: free thyroxine. TSH: thyroid-stimulating hormone. TRAb: thyrotropin receptor antibody. EOM: extraocular muscle. SIR: signal intensity ratio. T1RT: T1 relaxation time. T2RT: T2 relaxation time. FF: fat fraction

### Univariate and multivariate logistic regression analyses for activity staging of GO-affected eyes

Univariate and multivariate logistic regression analyses were performed, and the screening outcomes were shown in Table S5. The multivariate Logistic regression analysis identified the following parameters: EOM-FFmax (OR = 0.910, 95%CI: 0.810–1.021, *P* = 0.108); EOM-T1RTmean (OR = 1.003, 95%CI: 1.000–1.007, *P* = 0.061); EOM-T2RTmean (OR = 1.142, 95%CI: 1.055–1.235, *P* < 0.001); females (OR = 0.083, 95%CI: 0.011–0.614, *P* = 0.015); subclinical hyperthyroidism (OR = 0.057, 95%CI: 0.003–0.924, *P* = 0.044); hyperthyroidism (OR = 1.653, 95%CI: 0.285–9.576, *P* = 0.575); and hypothyroidism (OR = 0.121, 95%CI: 0.011–1.306, *P* = 0.082).

### Construction of a joint model for assessing the active status of GO-affected eyes

Multivariate logistic regression analysis was employed, with screening parameters (EOM-FFmax, EOM-T1RTmean, EOM-T2RTmean, sex, and thyroid functional status) serving as independent variables. In this study, three joint models were constructed to evaluate the active stage of GO-affected eyes. Specifically, joint Model 1 was founded on clinical parameters, joint Model 2 was based solely on MRI parameters, and joint Model 3 incorporated both MRI and clinical parameters (Table S6).

$$\eqalign{{\rm{Model}}\,1 & = 0.968 - 1.862 \times {\rm{females}} \cr& - 1.240 \times {\rm{subhyperthyroidism}} \cr& + {\rm{}}0.541 \times {\rm{hyperthyroidism}} \cr& + 0.284 \times {\rm{hypothyroidism}} \cr} $$

$$\eqalign{{\rm{Model}}\,2{\rm{}} & = - 17.092{\rm{}} - 0.075{\rm{}} \times {\rm{EOM-FFmax}} \cr& + 0.002{\rm{}} \times {\rm{EOMT-}}1{\rm{RTmean}} \cr& + 0.152 \times {\rm{EOMT-}}2{\rm{RTmean}} \cr} $$

$$\eqalign{{\rm{Model}}\,3{\rm{}} & = - 14.345{\rm{}} - 0.095{\rm{}} \times {\rm{EOM-FFmax}} \cr& + 0.003{\rm{}} \times {\rm{EOMT-}}1{\rm{RTmean}} + 0.132 \cr& {\rm{}} \times {\rm{EOMT-}}2{\rm{RTmean}} - 2.488{\rm{}} \cr& \times {\rm{females}} - 2.873{\rm{}} \times {\rm{subhyperthyroidism}} \cr& + 0.502{\rm{}} \times {\rm{hyperthyroidism}} \cr& - 2.114{\rm{}} \times {\rm{hypothyroidism}} \cr} $$

### Comparison of the diagnostic efficacy of activity status models for GO-affected eyes

DeLong’s test demonstrated that the differences in AUC values between Model 1 and Model 3, between Model 2 and Model 3, as well as between Model 3 and individual variables (EOM-T2RTmean, EOM-T1RTmean, and EOM-FFmax) were statistically significant (all *P* < 0.05) (Table S7).

**Fig. 2:**
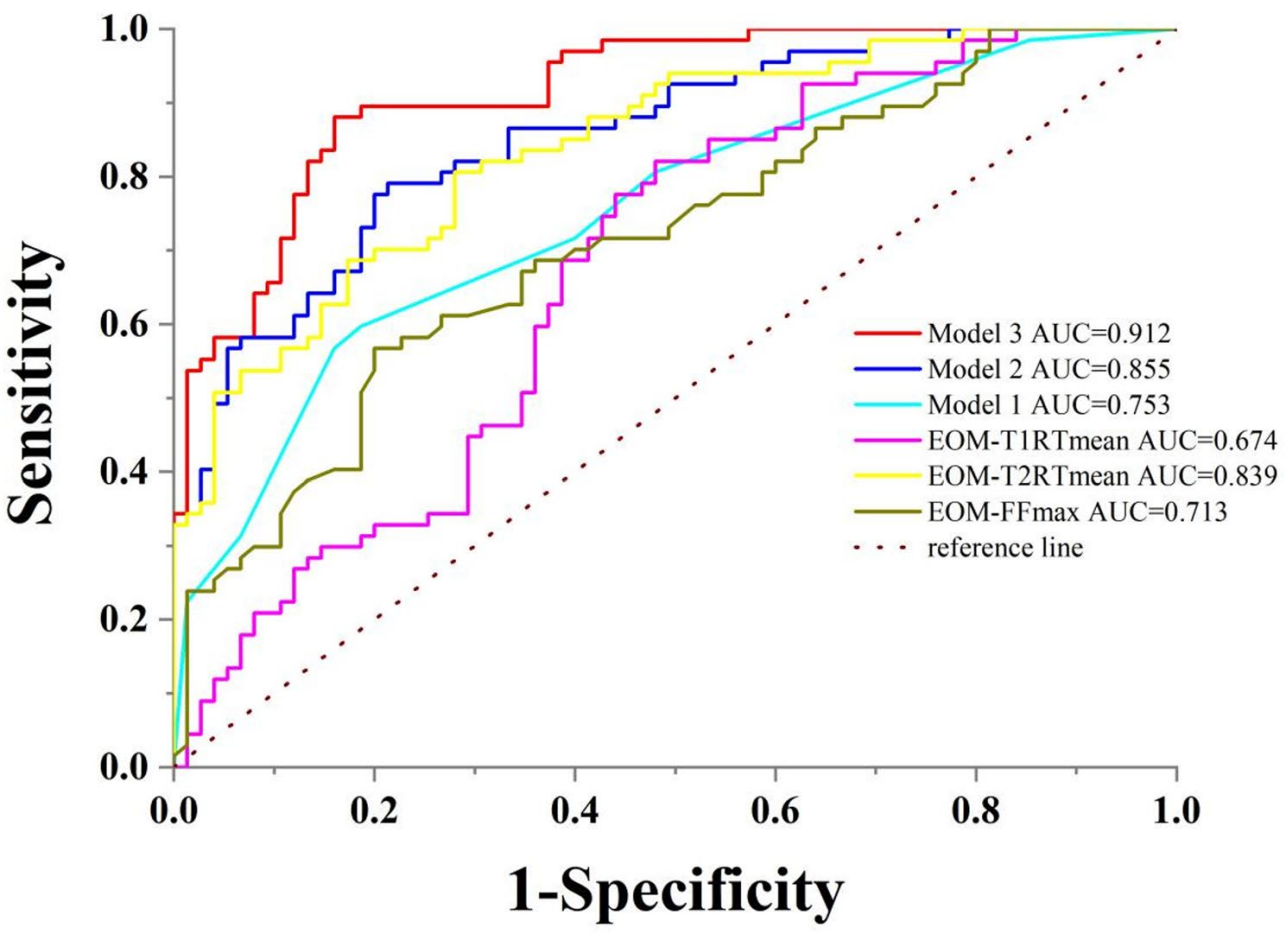
ROC curves for Model 1, Model 2, Model 3, and single MRI parameters in evaluating disease activity in GO-affected eyes. Model 1: sex + thyroid functional status. Model 2: EOM-FFmax + EOM-T1RTmean + EOM-T2RTmean. Model 3: EOM-FFmax + EOM-T1RTmean + EOM-T2RTmean + sex + thyroid functional status

Model 3 yielded the highest AUC, sensitivity, and specificity at 0.912, 88.06%, and 81.33%, respectively, for distinguishing between active and inactive GO groups (Table S8, Fig. 2).

### Validation of the joint model 3

Model 3 was subjected to internal validation using the leave-one-out method. The results indicated an AUC of 0.884 (95% CI: 0.832–0.937), a sensitivity of 85.1%, and a specificity of 76%. The calibration curves demonstrated good agreement between the probability predicted by Model 3 for a GO patient to be in the active group and the actual probability. Additionally, the H-L test yielded a P-value of 0.323, indicating good model fit (Fig. S3).

### Comparison of clinical and MRI parameters between mild and moderate–severe groups

Compared with the mild group, the moderate-severe group showed higher values for EOM-SIRmean, EOM-SIRmax, EOM-T1RTmean, EOM-T2RTmean, EOM-T2RTmax, and TRAb, while presenting lower values for EOM-FFmean, EOM-FFmax, and female proportion (all *P* < 0.05). Moreover, there was a statistically significant difference in thyroid functional status between the moderate-severe group and mild group (*P* = 0.002). No significant differences between the groups were observed in smoking history, disease duration, FT3, FT4, TSH, and EOM-T1RTmax (all *P* > 0.05) (Table 2, Fig. S4).

**Table 2:** Comparison of clinical and MRI parameters between mild and moderate–severe groups

Parameter	Mild group (*n* = 70)	Moderate-severe group (*n* = 72)	*P*
Sex (n, %)			< 0.001
Male	13 (19%)	41 (57%)	
Female	57 (81%)	31 (43%)	
Smoking (n, %)			0.136
+	8 (11%)	16 (22%)	
−	62 (89%)	56 (78%)	
Thyroid functional status (n, %)			0.002
Normal	33 (47%)	31 (43%)	
Subclinical hyperthyroidism	14 (20%)	2 (2.8%)	
Hyperthyroidism	17 (24%)	23 (32%)	
Hypothyroidism	6 (8.6%)	16 (22%)	
Disease duration (months)	6 (2, 12)	4 (2, 7)	0.195
FT3 (pg/mL)	5.11 (4.49, 7.96)	4.99 (4.23, 6.54)	0.410
FT4 (ng/dL)	17.80 (15.34, 22.64)	17.28 (14.52, 23.16)	0.189
TSH (µIU/mL)	0.53 (0.01, 1.69)	1.11 (0.01, 3.42)	0.081
TRAb (IU/L)	4.92 (2.98, 9.94)	10.22 (2.93, 21.56)	0.011
EOM-FFmean (%)	7.60 (5.30, 10.80)	4.90 (2.30, 6.70)	< 0.001
EOM-FFmax (%)	11.00 (8.00, 19.00)	7.00 (4.00, 10.00)	< 0.001
EOM-SIRmean	0.88 (0.80, 1.03)	1.20 (1.06, 1.32)	< 0.001
EOM-SIRmax	1.04 (0.93, 1.24)	1.42 (1.28, 1.56)	< 0.001
EOM-T1RTmean (ms)	1217.00 (1063.00, 1392.00)	1408.00 (1288.00, 1598.00)	< 0.001
EOM-T1RTmax (ms)	1620.00 (1461.00, 1745.00)	1727.00 (1484.00, 1885.00)	0.071
EOM-T2RTmean (ms)	86.00 (81.00, 96.00)	105.00 (96.00, 119.00)	< 0.001
EOM-T2RTmax (ms)	95.00 (89.00, 104.00)	122.00 (108.00, 139.00)	< 0.001

Note: P values are calculated from generalized mixed linear models. FT3: free triiodothyronine. FT4: free thyroxine. TSH: thyroid-stimulating hormone. TRAb: thyrotropin receptor antibody. EOM: extraocular muscles. SIR: signal intensity ratio. T1RT: T1 relaxation time. T2RT: T2 relaxation time. FF: fat fraction

### Univariate and multivariate logistic regression analyses for severity classification of GO-affected eyes

Univariate and multivariate logistic regression analyses were performed, and the screening outcomes were shown in Table S9. The multivariate logistic analysis identified the following parameters: EOM-FFmax (OR = 0.870, 95%CI: 0.747–1.013, *P* = 0.073), EOM-T1RTmean (OR = 1.005, 95%CI: 0.999–1.012, *P* = 0.113), EOM-T2RTmean (OR = 1.127, 95%CI: 1.023–1.241, *P* = 0.016), females (OR = 0.050, 95%CI: 0.001–1.875, *P* = 0.105), TRAb (OR = 1.082, 95%CI: 0.997–1.174, *P* = 0.060), subclinical hyperthyroidism (OR = 0.004, 95%CI: 0.000–0.729, *P* = 0.038), hyperthyroidism (OR = 0.920, 95%CI: 0.126–6.691, *P* = 0.934), and hypothyroidism (OR = 0.217, 95%CI: 0.012–3.877, *P* = 0.299).

### Construction of a joint model for assessing the disease severity of GO-affected eyes

Multivariate logistic regression analysis was employed, with screening parameters (EOM-FFmax, EOM-T1RTmean, EOM-T2RTmean, sex, thyroid functional status, and TRAb) serving as independent variables. In this study, three joint models were constructed to evaluate the severity of GO-affected eyes: joint Model 4 based on clinical parameters alone, joint Model 5 based on MRI parameters alone, and joint Model 6 integrating both MRI and clinical parameters (Table S10).

$$\eqalign{{\rm{Model}}\,4{\rm{}} & = {\rm{}}0.712 - {\rm{}}1.999{\rm{}} \cr& \times {\rm{females}} + {\rm{}}0.058{\rm{}} \times {\rm{TRAb}} \cr& - {\rm{}}2.587{\rm{}} \times {\rm{subhyperthyroidism}} \cr& {\rm{}} + {\rm{}}0.173{\rm{}} \times {\rm{hyperthyroidism}} \cr& + {\rm{}}0.681{\rm{}} \times {\rm{hypothyroidism}} \cr} $$

$$\eqalign{{\rm{Model}}\,5{\rm{}} & = - 249.381{\rm{}} + {\rm{}}0.009{\rm{}} \times {\rm{EOM-FFmax}} \cr& + {\rm{}}0.091{\rm{}} \times {\rm{EOMT-}}1{\rm{RTmean}} \cr& {\rm{}} + {\rm{}}1.382{\rm{}} \times {\rm{EOMT-}}2{\rm{RTmean}} \cr} $$

$$\eqalign{{\rm{Model}}\,6{\rm{}} & = - 15.292{\rm{}} - {\rm{}}0.139{\rm{}} \times {\rm{EOM-FFmax}} \cr& + {\rm{}}0.005 \times {\rm{EOMT-}}1{\rm{RTmean}} \cr& + {\rm{}}0.119{\rm{}} \times {\rm{EOMT-}}2{\rm{RTmean}} \cr& - {\rm{}}2.992{\rm{}} \times {\rm{females}} + {\rm{}}0.079{\rm{}} \times {\rm{TRAb}} \cr& {\rm{}} - {\rm{}}5.549{\rm{}} \times {\rm{subhyperthyroidism}} \cr& {\rm{}} - {\rm{}}0.084{\rm{}} \times {\rm{hyperthyroidism}} \cr& - {\rm{}}1.527{\rm{}} \times {\rm{hypothyroidism}} \cr} $$

### Comparison of the diagnostic efficacy of severity grading models for GO-affected eyes

DeLong’s test demonstrated that the differences in AUC values between Model 4 and Model 6, between Model 5 and Model 6, as well as between Model 3 and individual variables (EOM-T2RTmean, EOM-T1RTmean, and EOM-FFmax) were statistically significant (all *P* < 0.05) (Table S11).

**Fig. 3:**
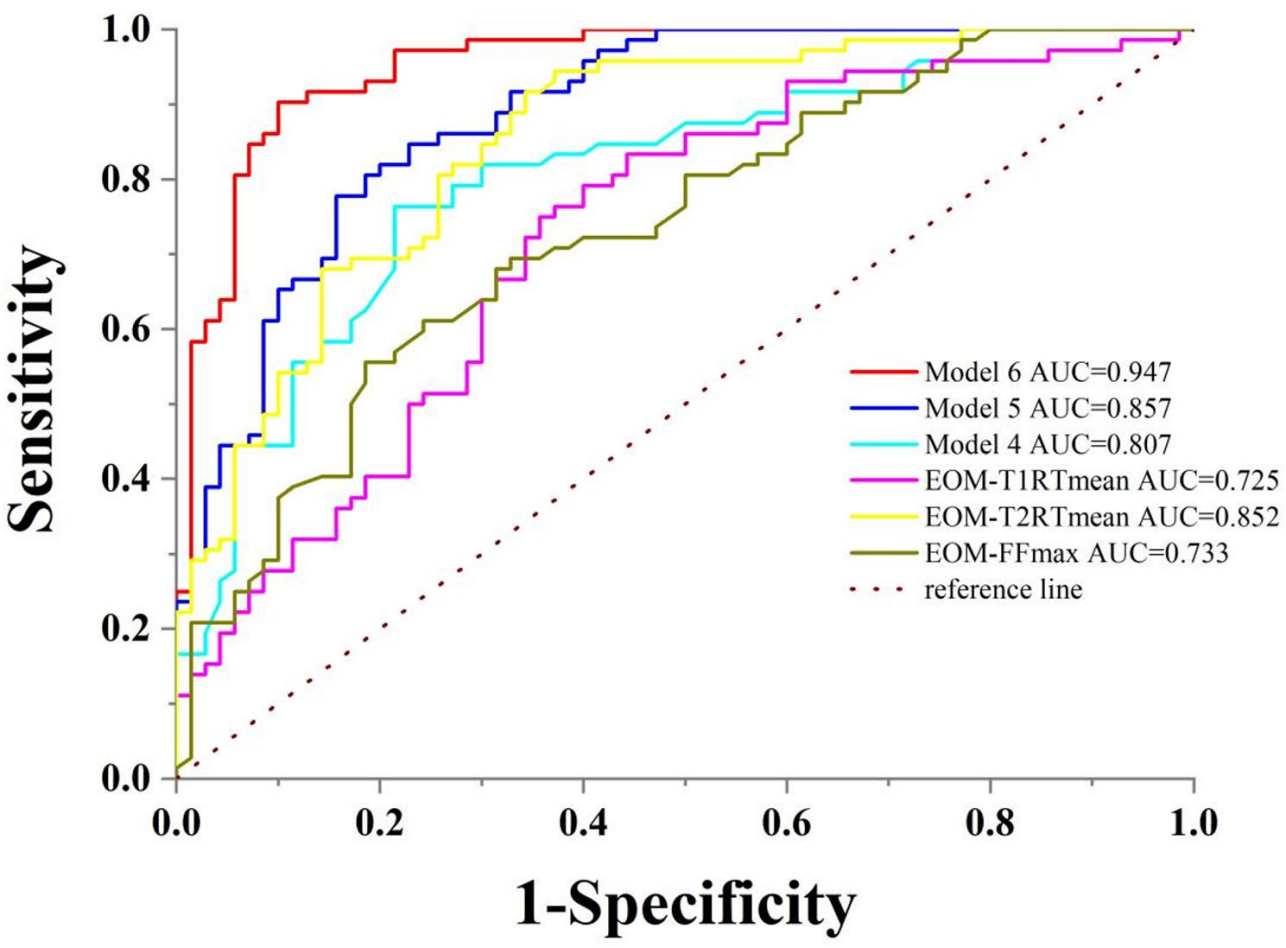
ROC curves for Model 4, Model 5, Model 6, and single MRI parameters for severity grading of GO-affected eyes. Model 4: sex + TRAb + thyroid functional status. Model 5: EOM-FFmax + EOM-T1RTmean + EOM-T2RTmean. Model 6: EOM-FFmax + EOM-T1RTmean + EOM-T2RTmean + sex + TRAb + thyroid functional status

Model 6 yielded the highest AUC and specificity at 0.947 and 92.86%, respectively, for distinguishing between the mild and moderate–severe groups (Table S12, Fig. 3).

### Validation of the joint model 6

Model 6 was subjected to internal validation using the leave-one-out method. The results indicated an AUC of 0.923 (95% CI: 0.878–0.969), a sensitivity of 90.2%, and a specificity of 87.1%. The calibration curves demonstrated good agreement between the probability predicted by Model 6 for a GO patient to be in the moderate-severe group and the actual probability. Additionally, the H-L test yielded a P-value of 0.076, indicating a good model fit (Fig. S5).

### Evaluation of disease activity and severity in go-affected eyes

**Fig. 4:**
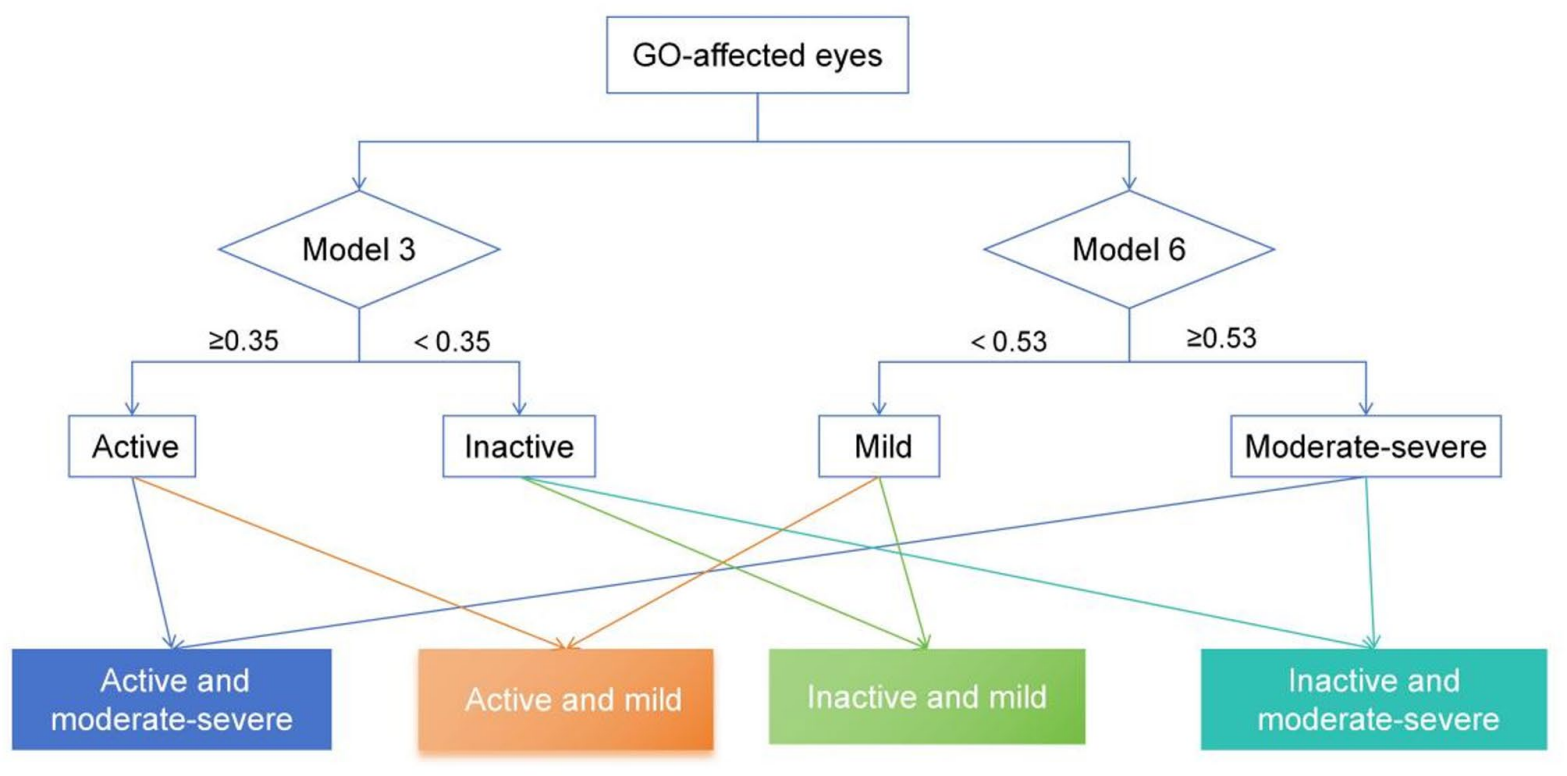
Flowchart for the assessment of disease activity and severity in GO-affected eyes. Model 3: EOM-FFmax + EOM-T1RTmean + EOM-T2RTmean + sex + thyroid functional status. Model 6: EOM-FFmax + EOM-T1RTmean + EOM-T2RTmean + sex + TRAb + thyroid functional status

Model 3 can be used to classify GO-affected eyes into active and inactive stages, whereas Model 6 categorizes them into mild and moderate-severe cases (Fig. 4). By integrating Models 3 and 6, GO-affected eyes can be further classified into four subtypes: mild in the active phase, moderate-severe in the active phase (Fig. S6), mild in the inactive phase (Fig. S6), and moderate-severe in the inactive phase.

## Discussion

Accurate grading and staging are critical for clinical treatment decisions. Although the CAS scoring and EUGOGO grading criteria are convenient, they lack objective quantitative parameters. This study explored the use of multiparametric quantitative MRI metrics combined with clinical metrics to evaluate GO eye activity and severity. The results showed that Model 3 for staging and Model 6 for grading performed best, with AUCs of 0.912 and 0.947, respectively. This finding suggests that combining MRI and clinical metrics significantly improves the assessment accuracy of GO-affected eyes.

The present study found that the moderate-severe GO group had a higher male percentage than the mild group, confirming males as an independent risk factor for moderate-severe GO, consistent with prior research [18, 19]. Male patients’ lower compliance with hyperthyroidism treatment may disrupt thyroid functional status, and as thyroid functional status correlates with GO severity [20, 21], this explains their more severe ocular disease. Similarly, the active GO group had a significantly higher male proportion, likely because female patients are more motivated to seek treatment for cosmetic reasons during the active phase [22] and estrogen may modulate inflammation [23].

T1 mapping technology directly measures tissue T1RT to reflect tissue properties [24]. It assesses edema, fibrosis, and fatty infiltration in conditions, such as chronic myocardial infarction, cirrhosis, and renal fibrosis [24, 25]. Here, the active group’s EOM-T1RTmean was significantly higher than the inactive group’s level, consistent with prior findings that active-phase inflammation/edema elevates EOM-T1RT, whereas inactive-phase fat infiltration reduces it [12, 26]. Moderate-severe cases also showed a higher EOM-T1RTmean than mild cases, with EOM-T1RTmean identified as an independent factor for GO eye severity, suggesting inflammatory edema in moderate-severe EOMs versus fatty infiltration in mild cases. However, there was no between-group difference in EOM-T1RTmax, probably because both active inflammatory edema and inactive fibrosis would increase EOM-T1RT values [12, 26].

T2 mapping directly measures tissue T2RT without reference tissue, offering simplicity and objectivity [10, 27]. It effectively reflects EOM inflammatory edema [8, 10]. In this study, EOM-T2RTmax and EOM-T2RTmean in the active group were significantly higher than those in the inactive group, with EOM-T2RTmean identified as an independent determinant of disease activity, which is consistent with prior findings [8, 10]. Active GO involves increased orbital glycosaminoglycan and hyaluronic acid synthesis, inducing EOM inflammatory edema and elevated T2RT, whereas inactive GO results in fibrotic EOMs with stable T2RT [10, 11]. Moderate-severe cases also exhibited higher EOM-T2RTmax and EOM-T2RTmean values than mild cases, with EOM-T2RTmean as an independent severity factor, further supporting inflammatory edema in moderate-severe EOMs. EOM edema/swelling disrupts the orbital venous/lymphatic circulation, causing proptosis, ocular motility dysfunction, and diplopia [28–30]. Increased inflammatory edema amplifies T2RT, exacerbates proptosis/diplopia, and is correlated with more severe GO.

The Dixon technique, the core of chemical shift water-fat separation, uses the chemical shift principle to precisely separate water and fat, yielding distinct signal images. With small inversion angles, multiple echo acquisitions, and T2* curve fitting, iron deposition interference is mitigated, ensuring accurate fat content maps [31, 32]. For SIR, this study found that EOM-SIRmean and EOM-SIRmax were higher in the active group than in the inactive group, consistent with prior research [15, 33]. EOM-SIR increased during the inflammatory edema stage and was higher in the moderate-severe group than in the mild group, providing a semiquantitative basis for evaluating GO. Regarding FF, EOM-FFmean and EOM-FFmax were lower in the active group than in the inactive group, which is in line with previous findings [10]. Fat content affects retrobulbar connective tissue remodeling. During GO’s active phase, ocular inflammation and inactivity cause fat deposition in the EOMs [34, 35]. As observed by Potgieser et al., mild GO patients experience EOM fatty infiltration [36], explaining why the severe group had lower EOM-FF values than the mild group in this study.

Previous studies have often assessed GO activity in a unidimensional manner. This study innovatively integrated multiparametric MRI metrics with clinical parameters, significantly improving the accuracy of GO activity and severity evaluation. For GO activity assessment, a model combining EOM-FFmax, EOM-T1RTmean, EOM-T2RTmean, sex, and thyroid functional status achieved an AUC of 0.912, sensitivity of 88.06%, and specificity of 81.33%, outperforming other models and prior staging methods [37, 15, 10]. For GO severity assessment, a model incorporating EOM-FFmax, EOM-T1RTmean, EOM-T2RTmean, sex, thyroid functional status, and TRAb achieved an AUC of 0.947 and a specificity of 92.86%, outperforming other models. These findings underscore the complementary roles of MRI and clinical parameters in the evaluation of GO severity. Specifically, FF quantifies EOM fat content [8, 10], T2RT reflects water content [8, 10, 11], and T1RT captures fat-water-fiber changes [12, 13]. Clinical markers such as sex, thyroid functional status, and TRAb correlate with GO progression [18, 19]. Integrating these parameters creates a joint model for the comprehensive assessment of GO activity and severity.

This study had several limitations. First, as this was a retrospective single-center study with a small sample size, it lacked subgroup analysis for vision-threatening cases; prospective multicenter large-sample RCTs are needed for validation. Second, MRI metrics relied on single-level 2D ROI measurements, which may not fully reflect the overall orbital tissue status in GO. Future studies should adopt 3D ROI analysis for a more accurate assessment. Third, the absence of histopathological “gold standard” confirmation necessitates clarifying the correlation between imaging parameters and pathological changes to improve the evaluation accuracy. Fourth, this study did not investigate the role of MRI metrics in predicting or monitoring GO treatment efficacy, which will be a key focus for future research.

## Conclusion

The FF, T1RT, and T2RT serve as quantifiable MRI metrics for evaluating GO activity and severity. The joint model integrating these multiparametric indices with clinical parameters improves assessment accuracy and provides a reliable quantitative basis for the development of individualized treatment plans for patients with GO.

## Electronic Supplementary Material

Below is the link to the electronic supplementary material.

Supplementary Material 1

## Data Availability

The datasets during and analyzed during the current study are available from the corresponding author upon reasonable request. All data generated or analyzed in this study are included in this published article.

## Abbreviations

GO: Graves’ orbitopathy
CAS: Clinical activity score
EUGOGO: European group on Graves’ orbitopathy
EOM: Extraocular muscle
MRI: Magnetic resonance imaging
SIR: Signal intensity ratio
T2RT: T2 relaxation time
T1RT: T1 relaxation time
FF: Fat fraction
FT3: Free triiodothyronine
FT4: Free thyroxine
TSH: Thyroid stimulating hormone
TRAb: Thyrotropin receptor antibody
ROI: Regions of interest
ICC: Intraclass or interclass correlation coefficients
ROC: Receiver operating characteristic
AUC: Under the curve
H-L: Hosmer and Lemeshow

## References

[1] Bartalena L, Piantanida E, Gallo D, et al. Epidemiology, Natural History, Risk Factors, and Prevention of Graves’ Orbitopathy. Front Endocrinol (Lausanne). 2020;11:615993. 33329408 10.3389/fendo.2020.615993 PMC7734282

[2] Lee ACH, Kahaly GJ. Pathophysiology of thyroid-associated orbitopathy. Best Pract Res Clin Endocrinol Metab. 2023;37(2):101620. 35181241 10.1016/j.beem.2022.101620

[3] Bartalena L, Kahaly GJ, Baldeschi L, et al. The 2021 European Group on Graves’ orbitopathy (EUGOGO) clinical practice guidelines for the medical management of Graves’ orbitopathy. Eur J Endocrinol. 2021;185(4):G43–67. 34297684 10.1530/EJE-21-0479

[4] Mourits MP, Koornneef L, Wiersinga WM, et al. Clinical criteria for the assessment of disease activity in Graves’ ophthalmopathy: a novel approach. Br J Ophthalmol. 1989;73(8):639–44. 2765444 10.1136/bjo.73.8.639 PMC1041835

[5] Gerding MN, Prummel MF, Wiersinga WM. Assessment of disease activity in Graves’ ophthalmopathy by orbital ultrasonography and clinical parameters. Clin Endocrinol (Oxf). 2000;52(5):641–6. 10792345 10.1046/j.1365-2265.2000.00973.x

[6] Prummel MF, Suttorp-Schulten MS, Wiersinga WM, et al. A new ultrasonographic method to detect disease activity and predict response to immunosuppressive treatment in Graves ophthalmopathy. Ophthalmology. 1993;100(4):556–61. 8479715 10.1016/s0161-6420(93)31607-6

[7] Luccas R, Riguetto CM, Alves M, et al. Computed tomography and magnetic resonance imaging approaches to Graves’ ophthalmopathy: a narrative review. Front Endocrinol (Lausanne). 2023;14:1277961. 38260158 10.3389/fendo.2023.1277961 PMC10801040

[8] Cheng J, Zhang X, Lian J, et al. Evaluation of activity of Graves’ orbitopathy with multiparameter orbital magnetic resonance imaging (MRI). Quant Imaging Med Surg. 2023;13(5):3040–9. 37179934 10.21037/qims-22-814 PMC10167472

[9] Higashiyama T, Iwasa M, Ohji M. Quantitative Analysis of Inflammation in Orbital Fat of Thyroid-associated Ophthalmopathy Using MRI Signal Intensity. Sci Rep. 2017;7(1):16874. 29203853 10.1038/s41598-017-17257-6 PMC5715090

[10] Li Z, Luo Y, Feng X, et al. Application of Multiparameter Quantitative Magnetic Resonance Imaging in the Evaluation of Graves’ Ophthalmopathy. J Magn Reson Imaging. 2023;58(4):1279–89. 36780178 10.1002/jmri.28642

[11] Liu P, Chen L, Wang QX, et al. Histogram analysis of T2 mapping for detecting early involvement of extraocular muscles in patients with thyroid-associated ophthalmopathy. Sci Rep. 2020;10(1):19445. 33173086 10.1038/s41598-020-76341-6 PMC7655798

[12] Ma R, Geng Y, Gan L, et al. Quantitative T1 mapping MRI for the assessment of extraocular muscle fibrosis in thyroid-associated ophthalmopathy. Endocrine. 2022;75(2):456–64. 34549377 10.1007/s12020-021-02873-0

[13] Zhu H, Zou M, Wu D, et al. Quantitative assessment of extraocular muscles in Graves’ ophthalmopathy using T1 mapping. Eur Radiol. 2023;33(12):9074–83. 37466707 10.1007/s00330-023-09931-3

[14] Bartley GB, Gorman CA. Diagnostic criteria for Graves’ ophthalmopathy. Am J Ophthalmol. 1995;119(6):792–5. 7785696 10.1016/s0002-9394(14)72787-4

[15] Chen L, Hu H, Chen HH, et al. Usefulness of two-point Dixon T2-weighted imaging in thyroid-associated ophthalmopathy: comparison with conventional fat saturation imaging in fat suppression quality and staging performance. Br J Radiol. 2021;94(1118):20200884. 33353397 10.1259/bjr.20200884 PMC7934300

[16] Kaichi Y, Tanitame K, Itakura H, et al. Orbital Fat Volumetry and Water Fraction Measurements Using T2-Weighted FSE-IDEAL Imaging in Patients with Thyroid-Associated Orbitopathy. AJNR Am J Neuroradiol. 2016;37(11):2123–8. 27365323 10.3174/ajnr.A4859 PMC7963797

[17] Zhai L, Luo B, Wu H, et al. Prediction of treatment response to intravenous glucocorticoid in patients with thyroid-associated ophthalmopathy using T2 mapping and T2 IDEAL. Eur J Radiol. 2021;142:109839. 34252869 10.1016/j.ejrad.2021.109839

[18] Lee MH, Chin YH, Ng CH, et al. Risk Factors of Thyroid Eye Disease. Endocr Pract. 2021;27(3):245–53. 33655885 10.1016/j.eprac.2020.11.011

[19] Oeverhaus M, Winkler L, Stähr K, et al. Influence of biological sex, age, and smoking on Graves’ orbitopathy - a ten-year tertiary referral center analysis. Front Endocrinol (Lausanne). 2023;14:1160172. 37082130 10.3389/fendo.2023.1160172 PMC10110835

[20] Allahabadia A, Daykin J, Holder RL, et al. Age and gender predict the outcome of treatment for Graves’ hyperthyroidism. J Clin Endocrinol Metab. 2000;85(3):1038–42. 10720036 10.1210/jcem.85.3.6430

[21] Ponto KA, S VDO-S, Elflein H, et al. [Healthcare relevant data from an interdisciplinary consultation for endocrine orbitopathy]. Ophthalmologe. 2020;117(11):1105–11. 32034469 10.1007/s00347-020-01050-4 PMC7644527

[22] Perros P, Crombie AL, Matthews JN, et al. Age and gender influence the severity of thyroid-associated ophthalmopathy: a study of 101 patients attending a combined thyroid-eye clinic. Clin Endocrinol (Oxf). 1993;38(4):367–72. 8319368 10.1111/j.1365-2265.1993.tb00516.x

[23] FitzPatrick AM. Is Estrogen a Missing Culprit in Thyroid Eye Disease? Sex Steroid Hormone Homeostasis Is Key to Other Fibrogenic Autoimmune Diseases - Why Not This One? Front Immunol. 2022;13:898138. 35784325 10.3389/fimmu.2022.898138 PMC9248759

[24] Fernandes JL, Rochitte CE. T1 mapping: technique and applications. Magn Reson Imaging Clin N Am. 2015;23(1):25–34. 25476671 10.1016/j.mric.2014.08.007

[25] Dekkers IA, Lamb HJ. Clinical application and technical considerations of T(1) & T(2)(*) mapping in cardiac, liver, and renal imaging. Br J Radiol. 2018;91(1092):20170825. 29975154 10.1259/bjr.20170825 PMC6319842

[26] Matsuzawa K, Izawa S, Kato A, et al. Low signal intensities of MRI T1 mapping predict refractory diplopia in Graves’ ophthalmopathy. Clin Endocrinol (Oxf). 2020;92(6):536–44. 32090348 10.1111/cen.14178

[27] Messroghli DR, Moon JC, Ferreira VM, et al. Clinical recommendations for cardiovascular magnetic resonance mapping of T1, T2, T2*, and extracellular volume: A consensus statement by the Society for Cardiovascular Magnetic Resonance (SCMR) endorsed by the European Association for Cardiovascular Imaging (EACVI). J Cardiovasc Magn Reson. 2017;19(1):75. 28992817 10.1186/s12968-017-0389-8 PMC5633041

[28] Bahn RS. Current Insights into the Pathogenesis of Graves’ Ophthalmopathy. Horm Metab Res. 2015;47(10):773–8. 26361262 10.1055/s-0035-1555762

[29] Wang Y, Patel A, Douglas RS. Thyroid Eye Disease: How A Novel Therapy May Change The Treatment Paradigm. Ther Clin Risk Manag. 2019;15:1305–18. 31814726 10.2147/TCRM.S193018 PMC6858302

[30] Wang Y, Smith TJ. Current concepts in the molecular pathogenesis of thyroid-associated ophthalmopathy. Invest Ophthalmol Vis Sci. 2014;55(3):1735–48. 24651704 10.1167/iovs.14-14002 PMC3968932

[31] Li G, Xu Z, Chen Y, et al. Longitudinal assessment of marrow fat content using three-point Dixon technique in osteoporotic rabbits. Menopause. 2016;23(12):1339–44. 27529463 10.1097/GME.0000000000000721

[32] Middleton MS, Haufe W, Hooker J, et al. Quantifying Abdominal Adipose Tissue and Thigh Muscle Volume and Hepatic Proton Density Fat Fraction: Repeatability and Accuracy of an MR Imaging-based, Semiautomated Analysis Method. Radiology. 2017;283(2):438–49. 28278002 10.1148/radiol.2017160606 PMC5410959

[33] Ge Q, Zhang X, Wang L, et al. Quantitative evaluation of activity of thyroid-associated ophthalmopathy using short-tau inversion recovery (STIR) sequence. BMC Endocr Disord. 2021;21(1):226. 34774035 10.1186/s12902-021-00895-3 PMC8590769

[34] Fang S, Zhang S, Huang Y, et al. Evidence for associations between Th1/Th17 hybrid phenotype and altered lipometabolism in very severe graves orbitopathy. J Clin Endocrinol Metab. 2020;105(6). 10.1210/clinem/dgaa124 32173759

[35] Wiersinga WM, Regensburg NI, Mourits MP. Differential involvement of orbital fat and extraocular muscles in graves’ ophthalmopathy. Eur Thyroid J. 2013;2(1):14–21. 24783034 10.1159/000348246 PMC3821503

[36] Potgieser PW, de Win M, Wiersinga WM, et al. Natural Course of Mild Graves Orbitopathy: Increase of Orbital Fat But Decrease of Muscle Volume With Increased Muscle Fatty Degeneration During a 4-Year Follow-Up. Ophthalmic Plast Reconstr Surg. 2019;35(5):456–60. 30882587 10.1097/IOP.0000000000001319

[37] Chen L, Chen W, Chen HH, et al. Radiological Staging of Thyroid-Associated Ophthalmopathy: Comparison of T1 Mapping with Conventional MRI. Int J Endocrinol. 2020;2020:2575710. 33144856 10.1155/2020/2575710 PMC7599391

