# Supplementary material for: Multiparametric MRI quantitative metrics for grading and staging graves’ ophthalmopathy
Source: BMC Med Imaging. 2026 May 9;26:319. doi: 10.1186/s12880-026-02398-w (PMC13326481; doi:10.1186/s12880-026-02398-w)
Supplement: Supplementary file 1 — Supplementary Material 1 [file 12880_2026_2398_MOESM1_ESM.docx]

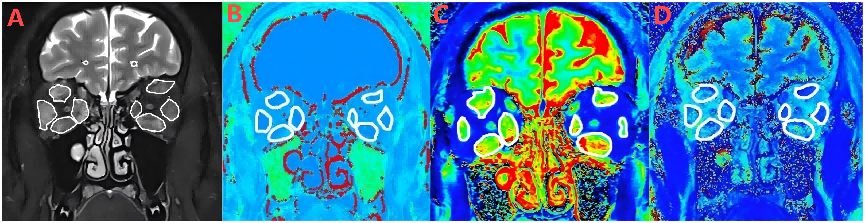


Fig S1 Schematic diagram of ROIs for EOM‑SIR, EOM‑FF, EOM‑T1RT, and EOM‑T2RT. In Panel A, SIR values were measured on coronal T2‑Dixon water-phase images. In Panel B, FF values were measured on coronal FF pseudo-color images. In Panel C, T1RT values were measured on coronal T1 mapping pseudo-color images. In Panel D, T2RT values were measured on coronal T2 mapping pseudo-color images


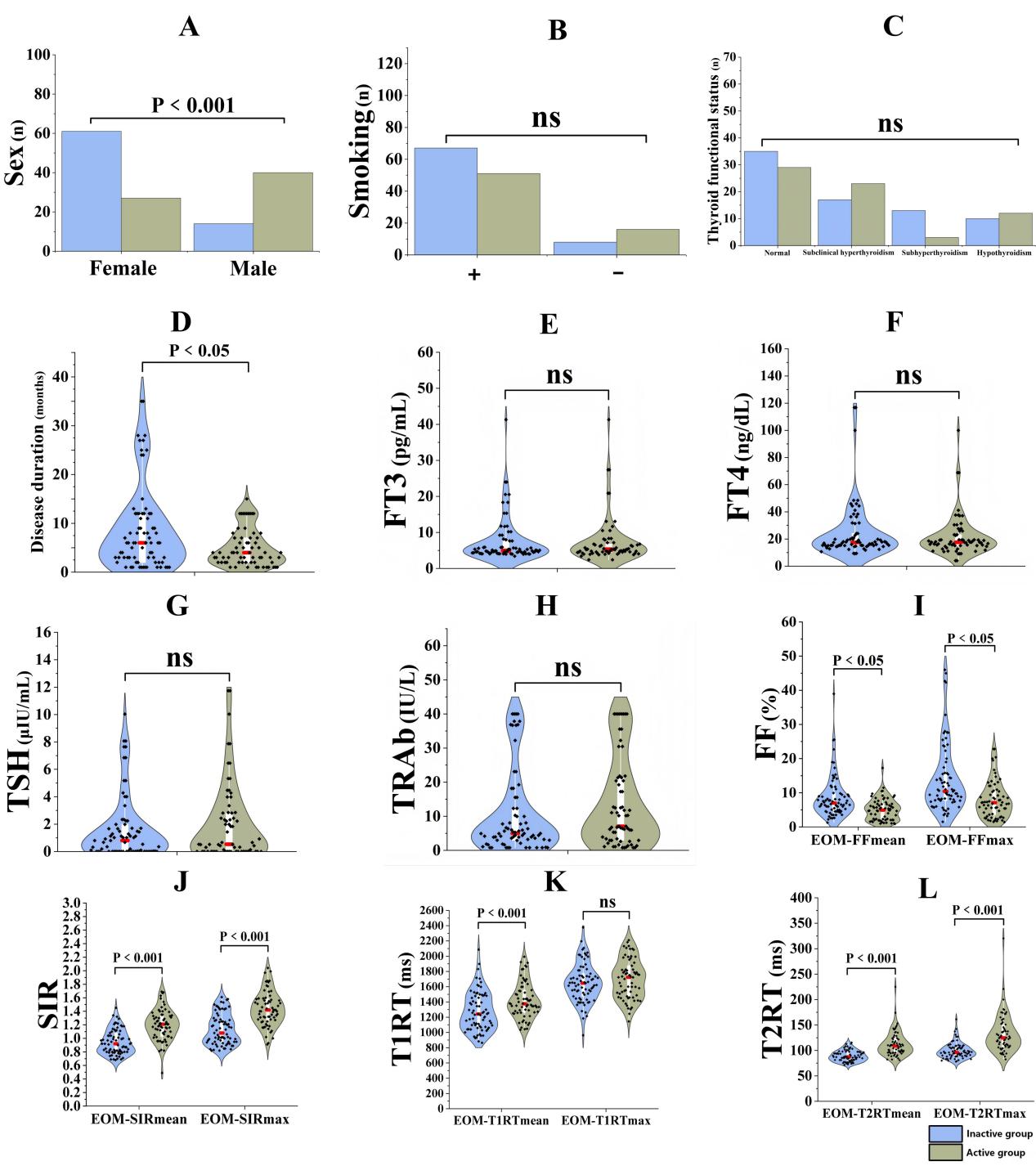


Fig S2 Comparison of clinical and MRI parameters between active and inactive GO-affected eyes. ns: no statistical difference. FT3: free triiodothyronine. FT4: free thyroxine. TSH: thyroid-stimulating hormone. TRAb: thyrotropin receptor antibody. EOM: extraocular muscles. SIR: signal intensity ratio. T1RT: T1 relaxation time. T2RT: T2 relaxation time. FF: fat fraction


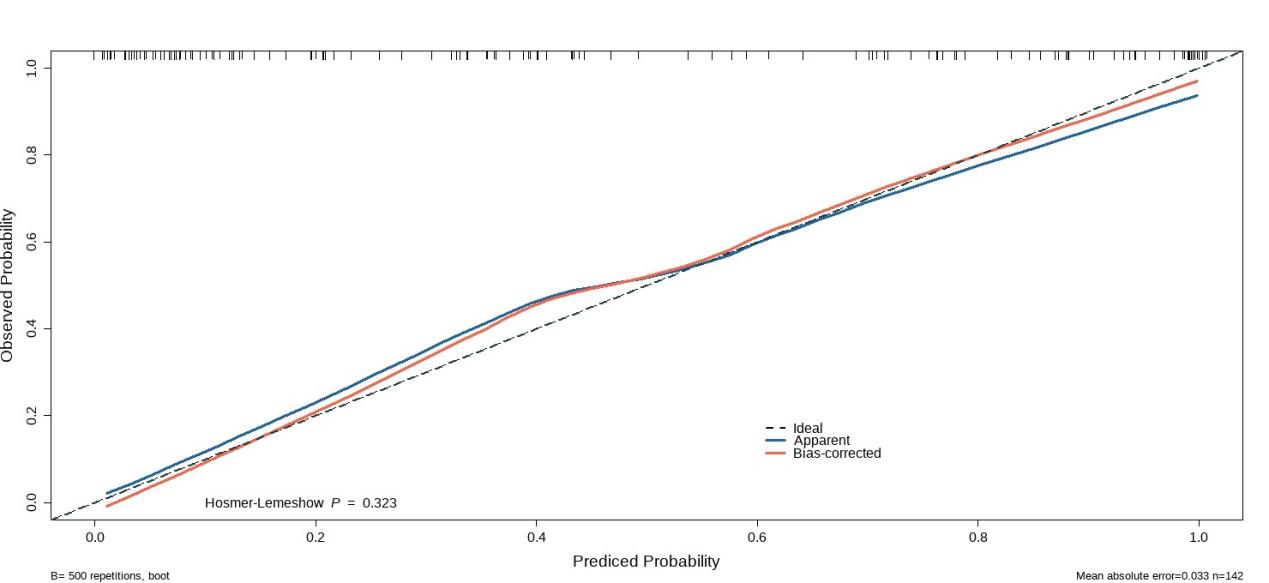


Fig S3 calibration curves of Model 3


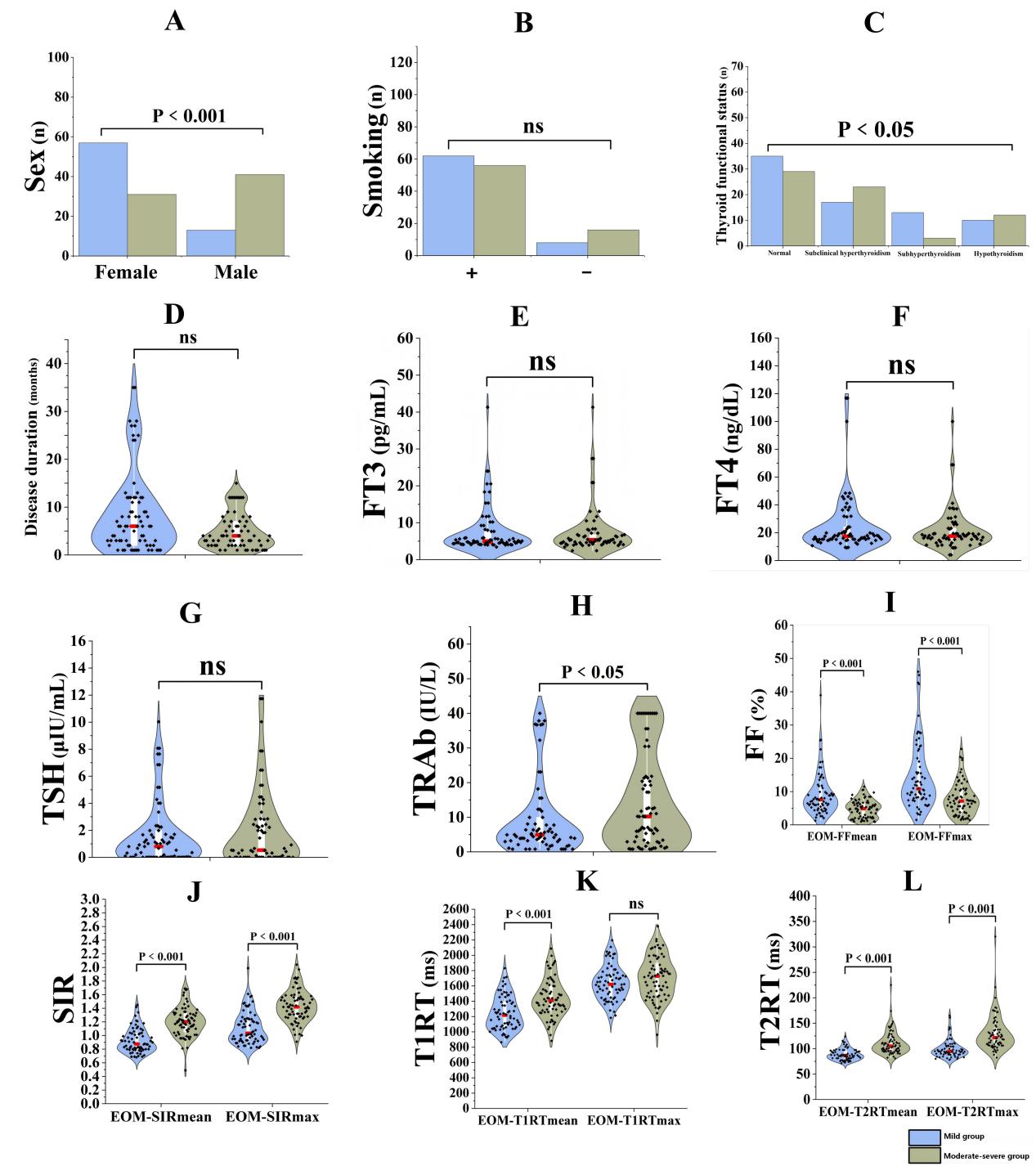


Fig S4. Comparison of clinical and MRI parameters between mild and moderate–severe GO-affected eyes. ns: no statistical difference. FT3: free triiodothyronine. FT4: free thyroxine. TSH: thyroid-stimulating hormone. TRAb: thyrotropin receptor antibody. EOM: extraocular muscles. SIR: signal intensity ratio. T1RT: T1 relaxation time. T2RT: T2 relaxation time. FF: fat fraction


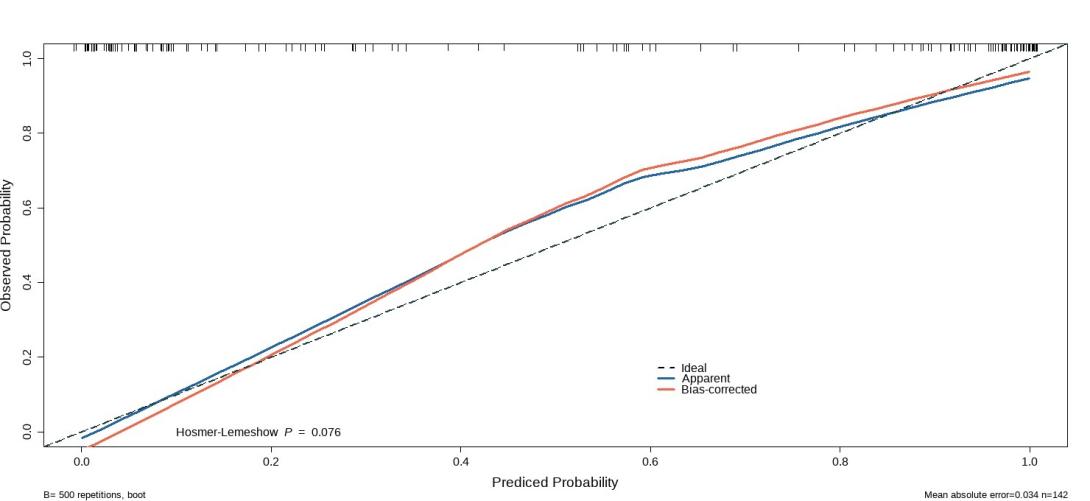


Fig S5 calibration curves of Model 6


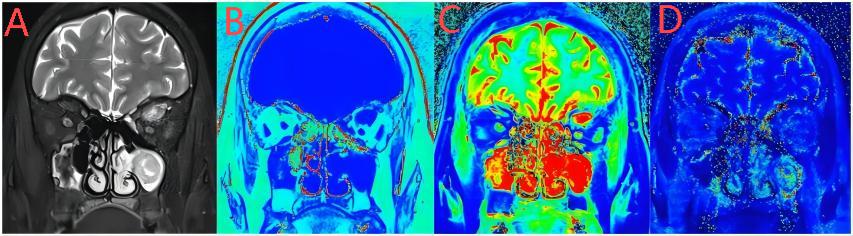


Fig S6 A 44-year-old hyperthyroid female (TRAb: 2.92 IU/L). According to CAS and EUGOGO criteria, the right eye was classified into the inactive mild group, and the left eye into the active moderate–severe group. (A) Coronal Dixon T2WI shows thickening of the left superior rectus muscle with inhomogeneous slightly high signal intensity. (B) On coronal FF mapping, the right eye EOM-FFmean was 3.56% and EOM-FFmax 8.3%; the left eye EOM-FFmean was 2.04% and EOM-FFmax 8.9%. (C) On coronal T1 mapping, the right eye EOM-T1RTmean was 1,058.49 ms, and the left eye EOM-T1RTmean was 1,542.1 ms. (D) On coronal T2 mapping, the right eye EOM-T2RTmean was 79.7 ms, and the left eye EOM-T2RTmean was 111.19 ms. As assessed by Model 3 and Model 6, the patient's right eye was inactive and mild, while the left eye was active and moderate–severe, consistent with the clinical evaluation

Table S1 Orbital MRI sequences and technical parameters

| **Sequence** | **T1WI** | **Dixon T2WI** | **Dixon T2WI** | **T1mapping** | **T2mapping** |
| --- | --- | --- | --- | --- | --- |
| **Scan direction** | Axial | Axial | Coronal | Coronal | Coronal |
| **TR (ms)** | 400 | 3120 | 3330 | 15 | 1140 |
| **TE (ms)** | 9.3 | 83 | 83 | 2.17 | 13.8, 27.6, 41.4, 55.2, 69 |
| **FOV (mm2)** | 180×180 | 180×180 | 180×180 | 180×180 | 180×180 |
| **Matrix** | 256×320 | 256×320 | 256×320 | 256×320 | 256×320 |
| **Slice thickness (mm)** | 3 | 3 | 3 | 3 | 3 |
| **No. of slices** | 15 | 15 | 16 | 22 | 16 |
| **Distance between slices (mm)** | 0.6 | 0.6 | 0.6 | 0.6 | 0.6 |
| **Flip angle** | 150° | 160° | 125° | 5°,26° | --- |
| **No. of excitations** | 2 | 1 | 1 | 1 | 1 |
| **Fat suppression method** | --- | water-lipid separation | water-lipid separation | --- | --- |
| **TA** | 2min14s | 2min14s | 2min23s | 2min31s | 3min48s |

Note: TR: repetition time; TE: echo time; FOV: field of view; TA: acquisition time

Table S2 Demographic and clinical characteristics of GO patients

| **Parameter** | **Result** (**n = 71)** |
| --- | --- |
| **Age (years)** | 21 ~ 75 |
| **Sex (n, %)** |  |
| **Male** | 27 (38%) |
| **Female** | 44 (62%) |
| **Smoking (n, %)** |  |
| **+** | 12 (17%) |
| **−** | 59 (83%) |
| **Thyroid functional status (n, %)** |  |
| **Normal** | 32 (45%) |
| **Subclinical hyperthyroidism** | 8 (11%) |
| **Hyperthyroidism** | 20 (28%) |
| **Hypothyroidism** | 11 (15%) |
| **Disease duration (months)** | 4 (2, 9) |
| **FT3 (pg/mL)** | 5.10 (4.40, 7.01) |
| **FT4 (ng/dL)** | 18.00 (15.00, 23.00) |
| **TSH (μIU/mL)** | 0.67 (0.01, 2.34) |
| **TRAb (IU/L)** | 6.00 (3.00, 18.00) |

Note: FT3: free triiodothyronine; FT4: free thyroxine; TSH: thyroid-stimulating hormone; TRAb: thyrotropin receptor antibody; EOM: extraocular muscle; SIR: signal intensity ratio; T1RT: T1 relaxation time; T2RT: T2 relaxation time; FF: fat fraction

Table S3 Intra-group ICC for multiparametric MRI quantitative metrics

| **Parameter** | **ICC** | |
| --- | --- | --- |
|  | **ICC** | **95% CI** |
| **EOM-FFmean (%)** | 0.962 | 0.935 ~ 0.977 |
| **EOM-FFmax (%)** | 0.937 | 0.896 ~ 0.963 |
| **EOM-SIRmean** | 0.949 | 0.916 ~ 0.969 |
| **EOM-SIRmax** | 0.925 | 0.878 ~ 0.955 |
| **EOM-T1RTmean (ms)** | 0.832 | 0.734 ~ 0.896 |
| **EOM-T1RTmax (ms)** | 0.735 | 0.593 ~ 0.833 |
| **EOM-T2RTmean (ms)** | 0.931 | 0.886 ~ 0.985 |
| **EOM-T2RTmax (ms)** | 0.825 | 0.723 ~ 0.891 |

Note: EOM: extraocular muscle; SIR: signal intensity ratio; T1RT: T1 relaxation time; T2RT: T2 relaxation time; FF: fat fraction

Table S4 Inter-group ICC for multiparametric MRI quantitative metrics

| **Parameter** | **ICC** | |
| --- | --- | --- |
|  | **ICC** | **95% CI** |
| **EOM-FFmean (%)** | 0.951 | 0.917 ~ 0.971 |
| **EOM-FFmax (%)** | 0.896 | 0.829 ~ 0.938 |
| **EOM-SIRmean** | 0.820 | 0.716 ~ 0.888 |
| **EOM-SIRmax** | 0.866 | 0.786 ~ 0.918 |
| **EOM-T1RTmean (ms)** | 0.832 | 0.734 ~ 0.896 |
| **EOM-T1RTmax (ms)** | 0.849 | 0.759 ~ 0.907 |
| **EOM-T2RTmean (ms)** | 0.816 | 0.710 ~ 0.886 |
| **EOM-T2RTmax (ms)** | 0.751 | 0.616 ~ 0.884 |

Note: EOM: extraocular muscle; SIR: signal intensity ratio; T1RT: T1 relaxation time; T2RT: T2 relaxation time; FF: fat fraction

Table S5 Univariate and multivariate logistic regression analyses for disease activity in GO-affected eyes

| **Parameter** | **Univariate Logistic** | | | **Multivariate Logistic** | | |
| --- | --- | --- | --- | --- | --- | --- |
|  | **OR** | **95% CI** | ***P*** | **OR** | **95% CI** | ***P*** |
| **EOM-FFmean** | 0.711 | 0.571 ~ 0.885 | 0.002 |  |  |  |
| **EOM-FFmax** | 0.836 | 0.742 ~ 0.941 | 0.003 | 0.910 | 0.810 ~ 1.021 | 0.108 |
| **EOM-SIRmean** | 7,040,414.717 | 136.555 ~ 362,984,896,488.259 | 0.004 |  |  |  |
| **EOM-SIRmax** | 24,315.979 | 111.290 ~ 5,312,827.123 | <0.001 |  |  |  |
| **EOM-T1RTmean** | 1.006 | 1.002 ~ 1.010 | 0.004 | 1.003 | 1.000 ~ 1.007 | 0.061 |
| **EOM-T1RTmax** | 1.002 | 0.999 ~ 1.004 | 0.207 |  |  |  |
| **EOM-T2RTmean** | 1.182 | 1.087 ~ 1.285 | <0.001 | 1.142 | 1.055 ~ 1.235 | <0.001 |
| **EOM-T2RTmax** | 1.100 | 1.053 ~ 1.149 | <0.001 |  |  |  |
| **Females** | 0.017 | 0.001 ~ 0.451 | 0.015 | 0.083 | 0.011 ~ 0.614 | 0.015 |
| **Smoking** | 8.159 | 0.508 ~ 130.989 | 0.138 |  |  |  |
| **Disease duration** | 0.787 | 0.942 ~ 0.946 | 0.007 |  |  |  |
| **Thyroid functional** |  |  | 0.383 |  |  | 0.048 |
| **Normal** | 1 |  |  | 1 |  |  |
| **Subclinical hyperthyroidism** | 0.191 | 0.012 ~ 3.008 | 0.239 | 0.057 | 0.003 ~ 0.924 | 0.044 |
| **Hyperthyroidism** | 1.933 | 0.293 ~ 12.762 | 0.494 | 1.653 | 0.285 ~ 9.576 | 0.575 |
| **Hypothyroidism** | 2.056 | 0.234 ~ 18.034 | 0.515 | 0.121 | 0.011 ~ 1.306 | 0.082 |
| **FT3** | 0.944 | 0.942 ~ 0.946 | <0.001 |  |  |  |
| **FT4** | 0.969 | 0.920 ~ 1.020 | 0.226 |  |  |  |
| **TSH** | 1.130 | 0.835 ~ 1.530 | 0.429 |  |  |  |
| **TRAb** | 1.028 | 0.959 ~ 1.102 | 0.431 |  |  |  |

Note: FT3: free triiodothyronine; FT4: free thyroxine; TSH: thyroid-stimulating hormone; TRAb: thyrotropin receptor antibody; EOM: extraocular muscle; SIR: signal intensity ratio; T1RT: T1 relaxation time; T2RT: T2 relaxation time; FF: fat fraction

Table S6 Models 1, 2, and 3 for predicting active stage in GO-affected eyes

| **Model** | **Parameter** | **Log(OR)** | **95% CI** | ***P*** |
| --- | --- | --- | --- | --- |
| **Model 1** |  |  |  |  |
|  | **Constant** | 0.968 | 0.258 ~ 1.738 |  |
|  | **Female** | -1.862 | -2.672 ~ 1.104 | <0.001 |
|  | **Thyroid functional status** |  |  |  |
|  | **Subclinical hyperthyroidism** | -1.240 | -2.868 ~ 0.116 | 0.095 |
|  | **Hyperthyroidism** | 0.541 | -0.331 ~ 1.430 | 0.227 |
|  | **Hypothyroidism** | 0.284 | -0.796 ~ 1.371 | 0.604 |
| **Model 2** |  |  |  |  |
|  | **Constant** | -17.092 | -28.487 ~ -5.697 | 0.003 |
|  | **EOM-FFmax** | -0.075 | -0.188 ~ 0.038 | 0.192 |
|  | **EOM-T1RTmean** | 0.002 | -0.002 ~ 0.006 | 0.264 |
|  | **EOM-T2RTmean** | 0.152 | 0.063 ~ 0.240 | <0.001 |
| **Model 3** |  |  |  |  |
|  | **Constant** | -14.345 | -23.560 ~ -5.131 | 0.002 |
|  | **EOM-FFmax** | -0.095 | -0.210 ~ 0.021 | 0.108 |
|  | **EOM-T1RTmean** | 0.003 | 0.000 ~ 0.007 | 0.061 |
|  | **EOM-T2RTmean** | 0.132 | 0.054 ~ 0.211 | <0.001 |
|  | **Female** | -2.488 | -4.488 ~ -0.487 | 0.015 |
|  | **Thyroid functional status** |  |  |  |
|  | **Subclinical hyperthyroidism** | -2.873 | -5.666 ~ -0.080 | 0.044 |
|  | **Hyperthyroidism** | 0.502 | -1.254 ~ 2.259 | 0.575 |
|  | **Hypothyroidism** | -2.114 | -4.495 ~ 0.267 | 0.082 |

Note: EOM: extraocular muscle; T1RT: T1 relaxation time; T2RT: T2 relaxation time; FF: fat fraction

Table S7 DeLong test of AUC values for Model 1, Model 2, and Model 3, as well as single‑parameter MRI in disease activity staging of GO-affected eyes

| **Model** | **dAUC** | **SE** | **95%CI** | **Z** | ***P*** |
| --- | --- | --- | --- | --- | --- |
| **Active vs. inactive groups** |  | | | | |
| **Model 1 - Model 2** | -0.102 | 0.049 | -0.198 ~ -0.006 | -2.081 | 0.037 |
| **Model 1 - Model 3** | -0.159 | 0.036 | -0.228 ~ -0.089 | -4.476 | <0.001 |
| **Model 1 - EOM-T2RTmean** | -0.086 | 0.049 | -0.181 ~ 0.009 | -1.774 | 0.076 |
| **Model 1 - EOM-FFmax** | 0.040 | 0.059 | -0.075 ~ 0.156 | 0.683 | 0.495 |
| **Model 1 - EOM-T1RTmean** | 0.080 | 0.066 | -0.049 ~ 0.209 | 1.212 | 0.226 |
| **Model 2 - Model 3** | -0.057 | 0.021 | -0.097 ~ -0.017 | -2.784 | 0.005 |
| **Model 2 - EOM-T2RTmean** | 0.016 | 0.013 | -0.010 ~ 0.041 | 1.203 | 0.229 |
| **Model 2 - EOM-FFmax** | 0.142 | 0.039 | 0.066 ~ 0.219 | 3.638 | <0.001 |
| **Model 2 - EOM-T1RTmean** | 0.181 | 0.041 | 0.101 ~ 0.262 | 4.394 | <0.001 |
| **Model 3 - EOM-T2RTmean** | 0.073 | 0.023 | 0.027 ~ 0.119 | 3.117 | 0.002 |
| **Model 3 - EOM-FFmax** | 0.199 | 0.041 | 0.119 ~ 0.280 | 4.859 | <0.001 |
| **Model 3 - EOM-T1RTmean** | 0.239 | 0.045 | 0.151 ~ 0.326 | 5.332 | <0.001 |
| **EOM-T2RTmean - EOM-FFmax** | -0.126 | 0.045 | -0.215 ~ -0.037 | -2.781 | 0.005 |
| **EOM-T2RTmean - EOM-T1RTmean** | -0.166 | 0.050 | -0.263 ~ -0.069 | -3.341 | <0.001 |
| **EOM-FFmax - EOM-T1RTmean** | 0.039 | 0.055 | -0.067 ~ 0.146 | 0.723 | 0.470 |

Note: Model 1: sex + thyroid functional status; Model 2: EOM-FFmax + EOM-T1RTmean + EOM-T2RTmean; Model 3: EOM-FFmax + EOM-T1RTmean + EOM-T2RTmean + sex + thyroid functional status

Table S8 The diagnostic efficacy of Model 1, Model 2, Model 3, and single MRI parameters in the assessment of disease activity in GO-affected eyes

| **Model** | **AUC** | **95% CI** | **Sensitivity** | **Specificity** | **Cut-off** | ***P*** |
| --- | --- | --- | --- | --- | --- | --- |
| **Model 1** | 0.753 | 0.674 ~ 0.833 | 59.70 | 81.32 | 0.42 | ﹤0.001 |
| **Model 2** | 0.855 | 0.785 ~ 0.908 | 80.60 | 77.33 | 0.35 | ﹤0.001 |
| **Model 3** | 0.912 | 0.871 ~ 0.959 | 88.06 | 81.33 | 0.35 | ﹤0.001 |
| **EOM-FFmax** | 0.713 | 0.629 ~ 0.797 | 56.72 | 80.00 | 0.60 | ﹤0.001 |
| **EOM-T1RTmean** | 0.674 | 0.585 ~ 0.726 | 82.09 | 52.00 | 0.30 | ﹤0.001 |
| **EOM-T2RTmean** | 0.839 | 0.776 ~ 0.903 | 80.60 | 72.00 | 0.34 | ﹤0.001 |

Note: Model 1: sex + thyroid functional status; Model 2: EOM-FFmax + EOM-T1RTmean + EOM-T2RTmean; Model 3: EOM-FFmax + EOM-T1RTmean + EOM-T2RTmean + sex + thyroid functional status

Table S9 Univariate and multivariate logistic regression analyses for the severity of GO-affected eyes

| **Parameter** | **Univariate Logistic** | | | **Multivariate Logistic** | | |
| --- | --- | --- | --- | --- | --- | --- |
|  | **OR** | **95% CI** | ***P*** | **OR** | **95% CI** | ***P*** |
| **EOM-FFmean** | 0.537 | 0.361 ~ 0.799 | 0.002 |  |  |  |
| **EOM-FFmax** | 0.792 | 0.683 ~ 0.918 | 0.002 | 0.870 | 0.747 ~ 1.013 | 0.073 |
| **EOM-SIRmean** | 356,799.595 | 188.168 ~ 676,555,224.102 | <0.001 |  |  |  |
| **EOM-SIRmax** | 30,553.228 | 81.845 ~ 11,405,748.915 | <0.001 |  |  |  |
| **EOM-T1RTmean** | 1.015 | 0.999 ~ 1.031 | 0.071 | 1.005 | 0.999 ~ 1.012 | 0.113 |
| **EOM-T1RTmax** | 1.003 | 1.000 ~ 1.006 | 0.072 |  |  |  |
| **EOM-T2RTmean** | 1.181 | 1.088 ~ 1.283 | <0.001 | 1.127 | 1.023 ~ 1.241 | 0.016 |
| **EOM-T2RTmax** | 1.092 | 1.048 ~ 1.138 | <0.001 |  |  |  |
| **Females** | 0.000 | 0.000 ~ 0.001 | <0.001 | 0.050 | 0.001 ~ 1.875 | 0.105 |
| **Smoking** | 8.695 | 0.265 ~ 285.395 | 0.225 |  |  |  |
| **Disease duration** | 0.749 | 0.593 ~ 0.946 | 0.015 |  |  |  |
| **Thyroid functional** |  |  | 0.049 |  |  | 0.002 |
| **Normal** | 1 |  |  | 1 |  |  |
| **Subclinical hyperthyroidism** | 0.010 | 0.000 ~ 0.931 | 0.046 | 0.004 | 0.000 ~ 0.729 | 0.038 |
| **Hyperthyroidism** | 2.143 | 0.088 ~ 52.146 | 0.640 | 0.920 | 0.126 ~ 6.691 | 0.934 |
| **Hypothyroidism** | 48.120 | 0.677 ~ 3,422.016 | 0.075 | 0.217 | 0.012 ~ 3.877 | 0.299 |
| **FT3** | 0.887 | 0.741 ~ 1.061 | 0.189 |  |  |  |
| **FT4** | 0.972 | 0.970 ~ 0.974 | <0.001 |  |  |  |
| **TSH** | 1.609 | 1.013 ~ 2.558 | 0.044 |  |  |  |
| **TRAb** | 1.078 | 0.987 ~ 1.176 | 0.093 | 1.082 | 0.997 ~ 1.174 | 0.060 |

Note: FT3: free triiodothyronine; FT4: free thyroxine; TSH: thyroid-stimulating hormone; TRAb: thyrotropin receptor antibody; EOM: extraocular muscles; SIR: signal intensity ratio; T1RT: T1 relaxation time; T2RT: T2 relaxation time; FF: fat fraction

Table S10 Models 4, 5, and 6 for predicting **the** severity of GO-affected eyes

| **Model** | **Parameter** | **Log(OR)** | **95% CI** | ***P*** |
| --- | --- | --- | --- | --- |
| **Model 4** |  |  |  |  |
|  | **Constant** | 0.712 | -0.053 ~ 1.539 | 0.077 |
|  | **Female** | -1.999 | -2.896 ~ -1.174 | <0.001 |
|  | **Thyroid functional status** |  |  | 0.008 |
|  | **Subclinical hyperthyroidism** | -2.587 | -4.618 ~ -1.014 | 0.003 |
|  | **Hyperthyroidism** | 0.173 | -0.757 ~ 1.106 | 0.714 |
|  | **Hypothyroidism** | 0.681 | -0.517 ~ 1.950 | 0.273 |
|  | **TRAb** | 0.058 | 0.024 ~ 0.097 | 0.001 |
| **Model 5** |  |  |  |  |
|  | **Constant** | -249.381 | -459.975 ~ -38.788 | 0.020 |
|  | **EOM-FFmax** | 0.009 | -2.308 ~ 2.325 | 0.994 |
|  | **EOM-T1RTmean** | 0.091 | 0.021 ~ 0.161 | 0.011 |
|  | **EOM-T2RTmean** | 1.382 | 0.207 ~ 2.557 | 0.021 |
| **Model 6** |  |  |  |  |
|  | **Constant** | -15.292 | -29.208 ~ -1.375 | 0.031 |
|  | **EOM-FFmax** | -0.139 | -0.291 ~ 0.013 | 0.073 |
|  | **EOM-T1RTmean** | 0.005 | -0.001 ~ 0.012 | 0.113 |
|  | **EOM-T2RTmean** | 0.119 | 0.022 ~ 0.216 | 0.016 |
|  | **Female** | -2.992 | -6.612 ~ 0.629 | 0.105 |
|  | **Thyroid functional status** |  |  | 0.044 |
|  | **Subclinical hyperthyroidism** | -5.549 | -10.783 ~ -0.316 | 0.038 |
|  | **Hyperthyroidism** | -0.084 | -2.068 ~ 1.901 | 0.934 |
|  | **Hypothyroidism** | -1.527 | -4.409 ~ 1.355 | 0.299 |
|  | **TRAb** | 0.079 | -0.003 ~ 0.161 | 0.060 |

Note: TRAb: thyrotropin receptor antibody; EOM: extraocular muscle; T1RT: T1 relaxation time; T2RT: T2 relaxation time; FF: fat fraction

Table S11 DeLong test of AUC values for Model 4, Model 5, and Model 6, as well as single‑parameter MRI in severity grading of GO-affected eyes

| **Model** | **dAUC** | **SE** | **95%CI** | **Z** | ***P*** |
| --- | --- | --- | --- | --- | --- |
| **Mild group vs. moderate-severe** |  | | | | |
| **Model 4 - Model 5** | -0.050 | 0.049 | -0.145 ~ 0.046 | -1.015 | 0.310 |
| **Model 4 - Model 6** | -0.140 | 0.032 | -0.203 ~ -0.077 | -4.373 | <0.001 |
| **Model 4 - EOM-T2RTmean** | -0.045 | 0.046 | -0.136 ~ 0.045 | -0.980 | 0.327 |
| **Model 4 - EOM-FFmax** | 0.074 | 0.055 | -0.034 ~ 0.182 | 1.350 | 0.177 |
| **Model 4 - EOM-T1RTmean** | 0.082 | 0.060 | -0.035 ~ 0.199 | 1.378 | 0.168 |
| **Model 5 - Model 6** | -0.091 | 0.027 | -0.143 ~ -0.038 | -3.400 | <0.001 |
| **Model 5 - EOM-T2RTmean** | 0.004 | 0.025 | -0.045 ~ 0.053 | 0.170 | 0.865 |
| **Model 5 - EOM-FFmax** | 0.124 | 0.044 | 0.038 ~ 0.209 | 2.832 | 0.005 |
| **Model 5 - EOM-T1RTmean** | 0.132 | 0.029 | 0.074 ~ 0.190 | 4.467 | <0.001 |
| **Model 6 - EOM-T2RTmean** | 0.095 | 0.026 | 0.043 ~ 0.147 | 3.595 | <0.001 |
| **Model 6 - EOM-T1RTmean** | 0.214 | 0.040 | 0.136 ~ 0.293 | 5.364 | <0.001 |
| **Model 6 - EOM-FFmax** | 0.222 | 0.041 | 0.141 ~ 0.303 | 5.386 | <0.001 |
| **EOM-T2RTmean - EOM-T1RTmean** | -0.127 | 0.049 | -0.224 ~ -0.031 | -2.581 | 0.010 |
| **EOM-T2RTmean - EOM-FFmax** | -0.120 | 0.046 | -0.209 ~ -0.030 | -2.609 | 0.009 |
| **EOM-T1RTmean -EOM-FFmax** | 0.008 | 0.053 | -0.096 ~ 0.111 | 0.150 | 0.881 |

Note: Model 4: sex + TRAb + thyroid functional status; Model 5: EOM-FFmax + EOM-T1RTmean + EOM-T2RTmean; Model 6: EOM-FFmax + EOM-T1RTmean + EOM-T2RTmean + sex + TRAb + thyroid functional status

Table S12 The diagnostic efficacy of Model 4, Model 5, Model 6, and single MRI parameters in the assessment of disease severity in GO-affected eyes

| **Model** | **AUC** | **95% CI** | **Sensitivity** | **Specificity** | **Cut-off** | ***P*** |
| --- | --- | --- | --- | --- | --- | --- |
| **Model 4** | 0.807 | 0.736 ~ 0.878 | 76.39 | 78.57 | 0.46 | ﹤0.001 |
| **Model 5** | 0.857 | 0.823 ~ 0.934 | 73.61 | 82.86 | 1.00 | ﹤0.001 |
| **Model 6** | 0.947 | 0.907 ~ 0.978 | 86.11 | 92.86 | 0.53 | ﹤0.001 |
| **EOM-FFmax** | 0.733 | 0.65 8 ~ 0.819 | 55.56 | 81.43 | 0.72 | ﹤0.001 |
| **EOM-T1RTmean** | 0.725 | 0.641 ~ 0.808 | 75.00 | 64.29 | 0.33 | ﹤0.001 |
| **EOM-T2RTmean** | 0.852 | 0.791 ~ 0.914 | 91.67 | 65.71 | 0.25 | ﹤0.001 |

Note: Model 4: sex + TRAb + thyroid functional status; Model 5: EOM-FFmax + EOM-T1RTmean + EOM-T2RTmean; Model 6: EOM-FFmax + EOM-T1RTmean + EOM-T2RTmean + sex + TRAb + thyroid functional status
